# Supplementary material for: Mental health in gay, lesbian and bisexual medical students
Source: PLOS Ment Health. 2025 Jan 16;2(1):e0000108. doi: 10.1371/journal.pmen.0000108 (PMC12798337; doi:10.1371/journal.pmen.0000108)
Supplement: Attachment — Submitted filename: response_to_rewiers_plosmentalhealth.pdf [file pmen.0000108.s002.pdf]

PMEN-D-24-00289
“Mental health and minority stress in LGBTQIA+ medical students”
PLOS Mental Health

Point-by-point responses to reviewers

For the convinience of the Editor and the reviewers we have retyped all suggestions and criticisms.

Dear Dr. Martins,

Thank you for submitting your manuscript to PLOS Mental Health. Sorry that the review process takes longer than usual as we had to invite a third reviewer to provide additional perspectives for the first round of review. After careful consideration of the reviewers' feedback, we feel that it has merit but does not fully meet PLOS Mental Health’s publication criteria as it currently stands. Therefore, we invite you to submit a revised version of the manuscript that addresses the points raised during the review process.

EDITOR:

When revising the manuscript, I recommend using Reviewer 2's comments as a reference point, as they provided comprehensive suggestions for improving various sections of the manuscript.

Reviewer 3 has serious concerns about the measures used, and I suggest reviewing the citations provided and explaining how you have addressed these limitations within the Brazilian context.

I would also appreciate seeing a paragraph in the introduction that elucidates the Brazilian context to better ground the findings of this study.

Response: We reviewed our manuscript according to all suggestions and criticisms of the reviewers. We also included in introduction a paragraph about Brazilian context

Reviewer #1:

Dear Authors,

Congratulations on your manuscript. It addresses a significant topic in scientific literature and provides valuable insights to the field. The manuscript is well-written and effectively elucidated with clear and informative tables. Additionally, it meets the journal's criteria comprehensively.

Response: Thank you for your comments.

Reviewer #2:

General comments

This is an interesting study that focuses on a population particularly susceptible to mental health problems, in whom it is necessary to have more information. However, the article has important limitations that unfortunately diminish its quality.

In general, the authors analyzed many variables with depressive symptoms, while it may be an objective for an article, is not entirely consistent with the title and objective stated in the study. For example, the title includes the concept of minority stress and in the Results Section I see only one variable related to minority stress (internalized stigma) and this has little prominence in the study.

Response: We agree that the main purpose of our study was mental health in gay, lesbian and bisexual medical students and not minority stress, that is a

component of mental health in this population. We changed the title of the manuscript to " Mental health in gay, lesbian and bisexual medical students”

If the study did not consider trans students because there were only 3 participants and did not include asexual, queer or intersex students, the article should be limited to the sexual orientations actually considered in the study. Therefore, including LGBTQIA+ in the title would be incorrect because not all of those sexual orientations, gender, and sex were represented.

Response: We agree and changed the title of the manuscript to " Mental health in gay, lesbian and bisexual medical students

A minor correction is that I believe the authors use the concept of “sexuality” when they mean “sexual orientation.” For example, the title of Table 2. Despite all the comments I point out below, I encourage the authors to improve their article to re submit it for evaluation to an academic journal.

Response: We agree that the correct is “sexual orientation” and not “sexuality”. We corrected the error in the title of Table 2 and reviewed the manuscript according to this suggestion.

Abstract

It is recommended to improve the reporting of the results by making a synthesis and a better integration of the results. This will improve their comprehension. It is recommended to include more information about the method in the abstract regarding the participants, instruments, and analyses used.

Response: We changed the Abstract to a better integration of the results, and included more information about the methods used in our study.

I believe it is not correct to state that the correlation of a prevalence will be examined since the authors conducted a study at the individual level. Rather, I believe that the authors seek to examine the association between mental health variables (e.g., depressive symptoms or symptom severity) with sociodemographic variables, quality of life, resilience, and internalized stigma. Prevalence is an epidemiological indicator with respect to a group/population,

therefore, the variable examined is not prevalence, but symptoms/severity of depression.

Response: We agree that we did not study “prevalence”. We aimed to study associations among variables concerning the medical students that participated in the study. We reviewed the manuscript and corrected this point.

Introduction

The first paragraph of the article corresponds to the relevance of the study, therefore, I recommend including it at the end of the introduction, before the objective.

Response: We moved the first paragraph to the end of the introduction, before the objective of the study.

An important aspect of this study is that it was conducted in Brazil. Given the importance of the social context in the minority stress model, I suggest incorporating briefly what is the mental health situation or other important information regarding the context in which LGBTQIA+ health university students live in Brazil, or of the Brazilian LGTBQIA+ population in general in case there is no data from the same population studied in the article.

Response: We included a brief description of data concerning the context of LGBTQIA+ university students in Brazil.

Materials and Methods

Study design and sample

The authors should mention the type of study used, which in this case would be a cross-sectional study.

Response: We included this information in methods section.

They should also include the number of participants, the recruitment method, number of participating universities, average age, inclusion/exclusion criteria, and any other data relevant to understanding who the participants are.

Response: We included more information concerning participant characteristics in Methods section.

I do not think it is necessary to repeat the variables measured in this section as they are mentioned in more detail below.

Response: We agree and modified the manuscript according to the suggestion.

Ethics statement

The ethics statement could be incorporated as part of the procedure, which the authors called Data Collection.

Response: We included the ethics statement, as suggested.

Data collection

I am not sure what the authors mean by “We considered the sample representative when the indications became repetitive and reached a plateau”. Since it is a study with a non-probability sample, the representativeness of the sample is a limitation of their study. There could be characteristics of people that influence who decide to answer the survey and this could constitute a bias. For example, it is possible that people with more symptoms decided to participate in your study and this may have influenced the high percentages of depression and anxiety found.

Response: We collected data until the people that was invited to participate in the study had already responded to the questionaire. We agree that a limitation of the study was that only people that were invited and agreed to participate in the study was evaluated. We included this consideration in the limitations of the study in Discussion section.

Instruments

I am not sure what is the reason why the authors assessed gender identity by two different variables (lines 154-156).

Response: We asked the participants to describe their gender identity (male, female, transgender, other) and sexual orientation (gay, lesbian, bisexual, other).

Also, I wonder what the usefulness of the body mass index variable in the context of the study is and how it was measured. Was it a self-report of BMI?

Response: We agree that BMI is not very relevant to the context of the study and excluded this information in the revised manuscript. BMI was calculated considering the self-report of weight and height.

There are variables called sociodemographic that are not precisely sociodemographic information (alcohol habits, BMI, academic performance, etc.).

Response: We changed the title of the table to "general characteristics of participants".

It is not clear if the internal consistencies of the instruments were obtained in the sample or are from other studies. It is recommended to incorporate the internal consistency of the instruments obtained in the sample.

Response: The internal consistency of the instruments was obtained with the data of our sample.

Is the symptom severity classification of the BDI and IDATE based on cut-off points? It is important to include this information.

Response: Yes, the symptom severity was based on previously described cut-off values of BDI and IDATE inventories. We included this information in the manuscrip and appropriate references.

It is recommended to include the references of the Brazilian adaptations of the instruments reported by the authors. It is not clear if they are already validated instruments or if they adapted them for this study. Were the items to assess quality of life created or adapted by the authors or are they based on measures

used in other studies? It is recommended to include this information and include the items. I do not think that readers are familiar with the concept of “quality of life in medical school”, so I believe that more information could be positive.

Response: All instruments used were previously translated and validated to Brazilian Portuguese. We included this information and the related references. The items used to assess quality of life were developed in previous published studies from our group. We changed methods to make these points clear.

I suggest that the instruments include what high scores on the scales mean, so that people can better understand the results. For example, higher scores on the RS-14 scale indicate greater resilience.

Response: We agree and modified the manuscript to explain the meaning of the scores.

Analysis

I am not sure what they mean by “According to Beck's Depression Inventory, the chi-square test was used”. I think it would be simpler to mention that t-test was used for continuous variables and chi2 for categorical variables. More information is needed on how they performed the analyses. For example, Table 4 compares quality of life with 3 levels of depression. T-test and logistic regression use binary variables, so it is not clear to me how they obtained this p-value. Also, in Table 5 there are different models and some with adjustment variables. It is not clear how these models were constructed, were all the models adjusted? what value of the sexual orientation variable is “adjusted for sexuality” and of gender is “adjusted for gender”?

Response: We modified the manuscript to better explain the statistical analysis of our data.

Results

What happened to the trans students, were they excluded from the analyses?

Response: Yes, trans students were excluded from the analyses.

Table 1 contains in the variable gender identity results for bisexual sexual orientation. I recommend including the information on the number of bisexual students by gender only in the text as it is confusing the way they included it in the table. It is the only variable that has a stratification. Given that it is a study with a small, non-random and, therefore, non-representative sample, I am not convinced by the use of the concept of prevalence. With the study conducted, could we be sure that 63.4% and 100% of the university population in LGB health in Brazil have depression and anxiety, respectively? I think it would be more appropriate to refer to these indicators as percentages.

Response: We agree that we cannot use the concept of prevalence, and we modified the text. We removed the information on the number of bisexual students by gender from the table

In Table 2 for the continuous variables SD and 95CI are used, it is suggested to use only one and that it be consistent throughout the study.

Response: We agree and removed information about 95%CI from table 2.

Table 3 has repeated information with Table 2 (e.g., symptoms of depression by gender and sexual orientation).

Response: We modified Table 3

Since this is a cross-sectional study, caution should be exercised with the interpretation of the results as a protective or risk factor. Maybe it is a translation error, but the authors mention “correlation” in some cases which I assume they mean “relation”. This is because they do not mention in the analyses that they have performed correlations.

Response: We agree that we did not study “correlation” and modified the text.

It is possible that the authors did not find statistically significant results given that the depression variable was recoded as a 3-category variable. This results in fewer people within each model, which clearly may influence lower statistical

power. Other alternatives are to use the continuous depression variable or to recode it into a binary variable for the regression models.

Discussion

The first paragraph should be a general summary of the results, without repeating percentages. This helps to have a general idea of the results of the study. Regarding the use of the concept of prevalence, it is clearer in the discussion that 63.4% could be overestimated compared to what is observed in the rest of the world. The same with having obtained 100% of moderate to severe anxiety symptoms.

Response: We revised the first paragraph of Discussion according to the suggestion of the reviewer. We observed that 63.4% of the participants of the study presented high values of depressive and anxiety symptoms, and that is probably higher than what has been observed in other countries.

It is not clear how the aspects mentioned in lines 315 to 317 contribute to minority stress. Moreover, no reference to other studies is made to affirm this. These aspects could also be factors that influence the mental health of non-LGB individuals, so I suggest improving this interpretation of the results.

Response: We modified Discussion and included references.

In the articles, the tables are not usually referred to in the discussion. The interpretation of the results between lines 386 and 393 is not clear. They mention that they found no differences, but then talk about that bisexual women were more vulnerable.

Response: We improved in Discussion the interpretation of the results.

The authors mention the number of participants as a strength; however, this number could be considered low. Why do they consider it a strength? The authors did not include limitations in the manuscript. Implications are mentioned in the conclusion, but these usually go at the end of the discussion.

Response: We included in Discussion the limitations of the study, including the criticisms of referees #2 and #3.

Reviewer #3:

The authors aim to discern the role of various sociodemographic characteristics on the presentation of depression, anxiety, social support, and resilience within a sexual minority medical student sample. My main concerns pertain to the use of the State-Trait Anxiety Inventory (STAI) that has poor psychometric properties as shown by previous studies and is not a measure of anxiety, and the questionable validity of all other scales used in the current sample as demonstrated by a nonsignificant correlation between the Beck Depression Inventory and the STAI. While I appreciate the authors' efforts to identify contributors of minority stress within sexual minority medical students, empirical models built upon questionable measures are unlikely to produce knowledge that will generalize to those outside of the current study sample. I realize it is a tall order to ask authors to validate each of these measures and deviate from the main purpose of the study but it is crucial that psychometric soundness of all measures used be properly ascertained and demonstrated.

Please see below for some of my suggestions that I offer in the hope that they may help increase the quality of this paper.

Response: All instruments used in our study were previously translated and validated to Brazilian Portuguese. We included this information in Methods Section. The internal consistency of the instruments was calculated using data obtained in our study and the Cronbach Alfa of the inventory is provided in Methods Section.

Gender versus sex assigned at birth: The authors use the terms referring to gender versus sex assigned at birth interchangeably, which should be revised so that the concept they are referring to is clear and consistent throughout the paper. In their sociodemographic table, they report percentages of people who identified as “male” versus “female” but then mention differences between men and

women. “male” and “female” do not refer to the social construct of gender and the paper needs to be revised to reflect this.

Response: We agree and revised the manuscript.

Please see the following guide on choosing terminology pertaining to gender provided by APA https://apastyle.apa.org/style-grammar-guidelines/bias-free-language/gender

Response: We revised the manuscript to use the terminology of APA.

The authors allude that they gave “other” gender and “other” sexual orientation in the Instruments section, but the tables imply they only provided “male” and “female” and “gay/lesbian” and “bisexual” as options. I think it would be helpful to revise and make it clear what categories were offered within the table or at least mention in a footnote, considering that people may respond differently based on options provided. For example, most researchers will treat being nonbinary, genderfluid, genderqueer, and gender non-conforming as discrete from being transgender, which is not the case and gender expansive individuals can identify as binary transgender, nonbinary and transgender, nonbinary and not transgender, etc. However, offering discrete categories would mask this intersection of identities within gender.

2. The authors should consider that many people who identify as plurisexual but not necessarily bisexual may have chosen bisexual because no other choices were given, which is often the case for individuals who identify as pansexual.

Response: Concerning the options in our questionnaire offered to the volunteers, we asked to describe themselves as man, women or other (specify) and gay, lesbian, bisexual or other (specify). We modified the manuscript to make this point clear.

2. The authors should consider that many people who identify as plurisexual but not necessarily bisexual may have chosen bisexual because no other choices were given, which is often the case for individuals who identify as pansexual.

Response: we added this consideration in Discussions, that people plurisexual or pansexual may have chosen to answer “bisexual” because of the choices offered in the questionnaire.

3. Did the authors exclude the transgender and pansexual identifying individuals they mention at the beginning of the Results section? Or were they just excluded from group comparisons?

Response: Since the number of individuals that considered themselves as transgender was small, they were excluded from all the analysis.

4. The limits of using the State-Trait Anxiety Inventory (STAI): It is a well-known fact that the STAI, especially the trait anxiety subscale, has serious psychometric limitations. Studies show that this measure does not assess for anxiety, but rather negative affectivity. Please refer to the following articles that make this very clear (as well as other articles that also further evidence this):

Bados, A., Gómez-Benito, J., & Balaguer, G. (2010). The state-trait anxiety inventory, trait version: does it really measure anxiety? Journal of personality assessment, 92(6), 560-567

Knowles, K. A., & Olatunji, B. O. (2020). Specificity of trait anxiety in anxiety and depression: Meta-analysis of the State-Trait Anxiety Inventory. Clinical psychology review, 82, 101928.

Accordingly, the authors will need to make considerable revisions in their manuscript so that the STAI is not referred to as an anxiety scale and thus, they could not have examined “anxiety” within this sample with it, unless they had other measures available (e.g., GAD-7).

Response: We agree and added a paragraph of limitation of the study: “In a review of 388 published studies of the Stait-Trait Anxiety Inventory (STAI-T), Knowles and Olatunji concluded that although this inventory was developed to measure an individual’s tendency to experience anxiety symptoms, there is a strong relationship between trait-anxiety measured by STAI-T with depressive

disorders and they proposed that STAI-T be considered a non-specific measure of negative affectivity rather than trait anxiety per se."

5. Relatedly, authors state "No significant correlation was found between depression and state anxiety" which is surprising given previous findings that suggest the opposite would hold: "Furthermore, anxiety and depressive symptom severity were similarly strongly correlated with the STAI-T (mean r = .59 – .61)." (Knowles & Olatunji, 2020). This makes me wonder if there are psychometric issues with the scales in this study's sample, which would not be surprising. The authors support their use of the measures they opted for based on Cronbach's alpha (which itself is a highly limited measure of internal consistency – for more information, please see: Rodriguez, A., Reise, S. P., & Haviland, M. G. (2016). Evaluating bifactor models: Calculating and interpreting statistical indices. Psychological methods, 21(2), 137.), but that does not speak towards construct validity – are these measures actually measuring depression and anxiety in this sample? I realize ascertaining this requires a lot more work and may be challenging to determine, but their results already suggest that the models they are running here may be inherently flawed, which warrants some investigation. It should not be surprising if the measures do not work the way they do in cisgender heterosexual samples, and authors can find that and show that, that itself is quite interesting and a valuable contribution of scientific knowledge for the field of minority mental health research. There is already some evidence that this may be the case:

Borgogna, N. C., Brenner, R. E., & McDermott, R. C. (2021). Sexuality and gender invariance of the PHQ-9 and GAD-7: Implications for 16 identity groups. Journal of Affective Disorders, 278, 122-130.

I wouldn't be surprised if these limitations of measurement apply to the remainder of all scales utilized in the current study, which makes it difficult to further comment on the discussion since all interpretations are assuming that all constructs were validly and reliably measured.

Response: The statement that "No significant correlation was found between depression and state anxiety" is wrong and was excluded from the final version

and it contradicts the paragraph that shows the opposite, a strong relation between these two measurements: “Severe trait anxiety was present in 72.7%, 56.9%, and 26.4% of the individuals with severe, moderate, and mild depression symptoms, respectively ($P<.001$).”

This is a very important point, that the scales used to evaluate anxiety and depression in minority groups may have a different cutoff value. We added the following paragraph in Discussion, about the limitations of the study:

“Borgogna et al demonstrated in college students that gender and sexual minorities can respond differently than cisgender/heterosexual men and women to anxiety and depression questionnaires and that the cutoff values for anxiety and depression may be different.”

6. Authors also do not provide how they determined severity of depression or “anxiety” using these scales – more information needs to be provided on how cutoffs were determined as well as support for those cutoff values.

Response: The cutoff values were based in previous studies and we included the references concerning these values.

7. Some edits are needed to make it clear the limits of generalizability; for example, the authors state “The prevalence of depression symptoms among LGBTQIA+ medical students was 63.4%, with 39.5% of the students experiencing severe symptoms”; rather, they should make it clear that this was within THEIR sample as this may make it appear that this is a sweeping generalization of all LGBTQ+ medical students.

Response: We agree that these values refer to our sample and cannot be considered “prevalence”. We corrected the manuscript to make this point clear.
