# Supplementary material for: Mental health in gay, lesbian and bisexual medical students
Source: PLOS Ment Health. 2025 Jan 16;2(1):e0000108. doi: 10.1371/journal.pmen.0000108 (PMC12798337; doi:10.1371/journal.pmen.0000108)
Supplement: Attachment — Submitted filename: response_to_reviewers_revision2.pdf [file pmen.0000108.s003.pdf]

PMEN-D-24-00289R1
“Mental health and minority stress in LGBTQIA+ medical students”
PLOS Mental Health

Point-by-point responses to reviewers

For the convenience of the Editor and the reviewers we have retyped all suggestions and criticisms.

Dear Dr. Martins,

Thank you for re-submitting your manuscript to PLOS Mental Health. Reviewer 1 has provided some additional comments following the earlier revision that your team made. After careful consideration, we feel that it has merit but does not fully meet PLOS Mental Health’s publication criteria as it currently stands. Therefore, we invite you to submit a revised version of the manuscript that addresses the points raised during the review process.

Please review your reference list to ensure that it is complete and correct. If you have cited papers that have been retracted, please include the rationale for doing so in the manuscript text, or remove these references and replace them with relevant current references. Any changes to the reference list should be mentioned in the rebuttal letter that accompanies your revised manuscript. If you need to cite a retracted article, indicate the article’s retracted status in the References list and also include a citation and full reference for the retraction notice.

Response: Thank you for your careful revision of our manuscript. We reviewed our reference list.

Have the authors made all data underlying the findings in their manuscript fully available (please refer to the Data Availability Statement at the start of the manuscript PDF file)?
Response: We sent with the revised manuscript all data that we used in our study.

6. Review Comments to the Author

Please use the space provided to explain your answers to the questions above. You may also include additional comments for the author, including concerns about dual publication, research ethics, or publication ethics. (Please upload your review as an attachment if it exceeds 20,000 characters)

Reviewer #2: I appreciate the opportunity to review this article. In this version of the manuscript, the authors have considerably improved the manuscript and the reporting of results, appropriately integrating the comments made in the first revision.

Some minor improvements to the paper are mentioned below.

General comment:

The aim of the study is different from what was done in the study, as the authors only analyze factors associated with depression among college students. As it is currently written it is implied that they will also analyze the relationship between some factors and anxiety.

Response: We revised the aim of the study to explain that our main purpose was to analyze factors associated with depression among gay, lesbian and bisexual medical students.

Abstract: Information about the measures used and the analysis performed is needed.

Response:

We included in the Abstract brief information about the measures used and the analysis performed.

Keywords

It may be useful to include LGB as a keyword.

Response: We included the suggested keyword.

Introduction

In the first paragraph it says "prevalence rate", but I think the authors mean "prevalence".

Response: We made this correction.

When the acronym LGBTQIA+ is mentioned for the first time, the meaning of each letter should be explained.

Response: We included the meaning of each letter in the first time we mentioned the acronym LGBTQIA+.

In the third paragraph the authors mention that LGBTQIA+ identity is a risk factor. I suggest modifying that sentence because it could be understood that the higher prevalence of mental health problems is due to an individual aspect of LGBTQIA+ people. For example, it could be mentioned that LGBTQIA+ people in different contexts exhibit worse mental health outcomes than their heterosexual and cisgender peers.

Response: We agree and followed your suggestion.

Results

Second paragraph, an SD has a % symbol.

Response: We corrected this error.

Third paragraph, I recommend when mentioning table 2 to say something similar to the title of table 2. As written in the manuscript it is not clear. It is mentioned that "Women exhibited higher overall quality of life scores than men did (P=.043).". However, in the table it is mentioned that this difference is not statistically significant.

Response: We made all the suggested corrections. The difference in quality of life between men and women was statistically significant. We corrected table 2.

In some parts of the results section the information in the tables is repeated in the text (for example, the means and percentages). I recommend improving the reporting of results by avoiding repeating information when it is not necessary, since the information is already in the table.

Response: We made the suggested change in the results section.

I recommend avoiding the use of terms such as protective factor or risk factor, as this is a cross-sectional study. Concepts such as protective/risk factor may imply causal relationships.

Response: We agree and suppressed the use of “protective” or “risk factor”, that are not appropriate to cross sectional studies.

It says “logistic regression”, but the authors used multinomial logistic regression. It repeats again in the discussion.

Response: We made this correction in the revised manuscript.

In the last paragraph, I did not understand what the authors mean by the sentence beginning with: “Other differences include living with friends instead of living alone or with family,…”

Response: We changed the manuscript to better explain this paragraph.

Discussion

The first paragraph should be a summary of the main results of the article. In the second paragraph, the authors mention different factors to explain the high presence of depression. It is not clear why these factors could increase minority stress. I personally believe that the results could account for the relationship between the presence of such general stressors (affecting all medical students)

and minority stressors (specific to the LGB group) (as states the minority stress model). On the other hand, it may be that the general factors are particularly influential on the LGB group. For example, the stress associated with entering the job market might be higher in LGB individuals due to expectations of rejection. Therefore, I recommend making more explicit what the authors mean by these factors that affect medical students increase minority stress.
The authors mentioned: “Additionally, our study revealed that gay, lesbian and bisexual students living alone or with their families experienced more depression symptoms than did those living with friends, a distinction not observed in the general population of students45, 54.” This is an interesting result that the authors could discuss further. Could it be related to students living at home having more family conflicts due to being part of the LGB community? are students living with friends from high socioeconomic status, so they have less mental health problems?

Response: We also believe that there is a relationship between the presence of such general stressors, that affect all medical students and minority stressors (specific to the LGB group), as stated in the minority stress model. We added a comment on the paragraph. We also believe that students living with their families can be exposed to more conflicts due to being LGB and sometimes they choose to live with friends, but there is no influence of the socioeconomic status of the friends. We changed the paragraph.

The authors mentioned: “Participation in sports associations was associated with lower levels of depression than engaging only in political activities.” However, they did not identify students that participate in political activities (the variable is coded sports/other).

Response: The majority of associations that were not for sport competitions were associations that include political activities. We changed the manuscript to make this point clear.

It is not clear what the authors mean by “in our study we only observed poorer perceptions of academic performance.”

Response: We changed the paragraph to better explain this observation.

It is not recommended to mention the tables in the discussion.

Response: We made the suggested changes.

Tables

Table 1 needs a first row with headings. P values could be included, even if they are not lower than 0.05. There is now one result that is greater than 0.05, but it is not marked as NS.

Response: We added a first row in table 1 with headings and included P values in all tables.

Table 2 in severe state anxiety for man, the numbers don't match.

Response: We corrected the error.

Table 4 has percentages by row (e.g. academic performance) and by column (trait anxiety), which is confusing. I recommend reporting percentages by row or column, but not the two.

Response: We corrected the percentages of academic performance and now report all percentages by column.

Table 5. Multinomial logistic regression does not estimate OR, but RRR. The authors need to correct this. This table is very long. The authors could make adjustments to reduce it. For example, it is not necessary to include the "No symptoms" column. It is recommended to include the RRR, CI, and p value in different columns (not in the same cell). I think the authors changed the order of the adjusted models a and b, because it says adjusted for sexual orientation and this variable appears in the model. The same for the models adjusted for gender.

Response: We corrected the error in Table 5, thank you for your careful revision of the manuscript. We excluded the column of “no symptoms”, as suggested, and in the legend reported that the group with no symptoms was used as reference in the regressions. We did not separate OR, CI and P values in different columns to not increase the size of the table. We kept OR instead of RRR because many authors recommend to report the results of multinomial logistic regressions in odds ratios.
