# Supplementary material for: Mental health in gay, lesbian and bisexual medical students
Source: PLOS Ment Health. 2025 Jan 16;2(1):e0000108. doi: 10.1371/journal.pmen.0000108 (PMC12798337; doi:10.1371/journal.pmen.0000108)
Supplement: Attachment — Submitted filename: point_by_point_response.pdf [file pmen.0000108.s004.pdf]

PMEN-D-24-00289R1
“Mental health and minority stress in LGBTQIA+ medical students”
PLOS Mental Health

Point-by-point responses to suggestions of the academic editor

For the convenience of the Editor we have retyped all suggestions and criticisms.

1. Include subheadings in your abstract: Introduction, Methods, Results, and Conclusion.

Response: We included the subheadings in the Abstract.

2. Italicize subheadings in different parts of the paper to make it clear to the production editor that these are subheadings.

Response: All subheadings in Introduction and Methods were italicized.

3. Edit this sentence on page 9 to “The analyses were performed using IBM SPSS Statistics Version 22.”

Response: We modified the sentence as suggested.

4. The character ‘P’ for p-value on page 10 should be italicized.

Response: we italicized the character “P” for p-value.

5. Edit this sentence on page 13 to “It is plausible that there are general stressors affecting all medical students, as well as minority stressors specific to the LGB group, as outlined in the minority stress model.”

Response: We modified the sentence on page 13 as suggested.

6. Edit this sentence on page 16 to “Another limitation of our study is that the cut off values of the depression inventory have not been established for sexual and gender minority populations.” There was a typo with ‘stablished’.

Response: We made this change.

7. The odds ratios in Table 5 should be rounded to two decimal places.”

Response: We rounded all values of odds ratios to two decimal places in Table 5.

8. I recommend another thorough read-through of the article by a professional editor, if possible.

Response: We sent our manuscript for another English revision by the American Journal Experts (AJE).

The new manuscript “with track changes” has both your suggestions and the suggestions made by the AJE (all underlined).
